# Non-Invasive Brain Delivery of G4 70/30 PAMAM Dendrimers via the Intranasal Route

**DOI:** 10.1021/acschemneuro.5c00787

**Published:** 2026-03-10

**Authors:** Nadia Allahyarzadeh Khiabani, Avery Russel-Jay Elmhirst, Osheen Dubey, Arjun Poudel, Mayank Singh, Hannah Monville, Andrew Sahouri, Yousef Alani, Douglas Swanson, Gary L. Dunbar, Julien Rossignol

**Affiliations:** † Field Neurosciences Institute Laboratory for Restorative Neurology, Central Michigan University, Mount Pleasant, Michigan 48858-3803, United States; ‡ Program in Neuroscience, Central Michigan University, Mount Pleasant, Michigan 48858-3803, United States; § College of Medicine, Central Michigan University, Mount Pleasant, Michigan 48858-3803, United States; ∥ Department of Psychology, Central Michigan University, Mount Pleasant, Michigan 48858-3803, United States; ⊥ Department of Chemistry and Biochemistry, Central Michigan University, Mount Pleasant, Michigan 48858-3803, United States; # Department of Biochemistry, Cellular and Molecular Biology, 5649 Central Michigan University, Mount Pleasant, Michigan 48858-3803, United States; ¶ The National Dendrimer & Nanotechnology Center, Mount Pleasant, Michigan 48858-3803, United States

**Keywords:** PAMAM dendrimer, intranasal, blood–brain barrier, drug delivery, nanocarriers, central nervous system targeting

## Abstract

Effective treatment of neurological disorders remains a major clinical challenge due to the restrictive nature of the blood–brain barrier (BBB), which limits the entry of most therapeutic agents into the central nervous system (CNS). In this study, we investigated the in vivo biodistribution, safety, and CNS targeting ability of fourth generation (G4) PAMAM dendrimers, containing 70% hydroxyl and 30% amine surface groups (G4 70/30) following intranasal administration in C57BL/6J mice. Male and female mice were administered daily intranasal doses of Cy5.5-labeled G4 70/30 PAMAM dendrimers for 4 weeks, while control animals received Hank’s balanced salt solution (HBSS; Gibco, Waltham, MA, USA). Whole-body fluorescence imaging was conducted weekly to assess biodistribution, followed by organ extraction and fluorescence microscopy to evaluate tissue-level accumulation. The results revealed substantial accumulation of dendrimers in the brain, with no signs of toxicity in major organs including the lungs, livers, and kidneys. Notably, male mice exhibited significantly higher fluorescent intensity in the brain compared to females. These findings support the safety and effectiveness of G4 70/30 PAMAM dendrimers for intranasal delivery and highlight their potential as carriers for CNS-targeted therapies, including drugs and nucleic acids, in the treatment of neurological disorders.

## Graphical abstract

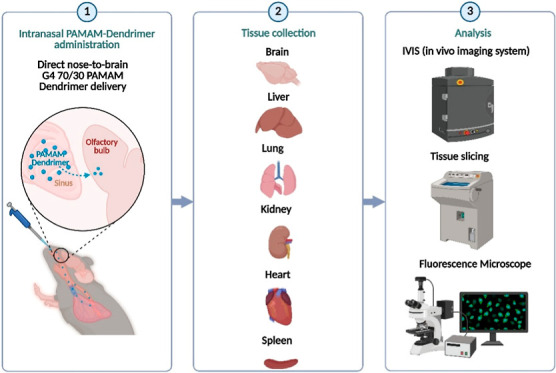

## 1 Introduction

In the realm of drug development, drug delivery systems can be thought of as developed solutions for the controlled distribution of targeted or nontherapeutic substances in recent decades.^1,2^

Intranasal administration takes advantage of the unique anatomical and functional connection between the nasal mucosa and the brain. Drugs deposited in the upper nasal cavity, particularly within the olfactory region, can bypass systemic circulation and enter the central nervous system (CNS) through two primary routes: transport along olfactory nerve fibers into the olfactory bulb, and diffusion via the trigeminal nerve to deeper brain regions. This pathway enables direct access to the CNS while circumventing the blood–brain barrier (BBB) which is one of the most formidable barriers in neuropharmacology.^3^ The BBB is a highly selective and dynamic interface that separates the peripheral blood circulation from the brain parenchyma. Its primary role is to maintain CNS homeostasis by preventing the entry of pathogens, immune cells, and neurotoxic plasma components, while tightly regulating the transport of ions, nutrients, and metabolites required for neuronal activity. Structurally, the BBB is formed by a monolayer of specialized endothelial cells joined by tight junctions, supported by pericytes, astrocytic end-feet, and the surrounding extracellular matrix. While essential for protecting 86 billion neurons of the brain, this highly regulated barrier also poses a major obstacle to the delivery of therapeutic macromolecules, biologics, and most small-molecule drugs.^4–6^ Given these challenges, the intranasal route has emerged as a promising alternative for CNS drug delivery, offering a noninvasive strategy that avoids systemic metabolism and provides targeted access to the brain. The nasal cavity plays a dual role in respiratory protection and sensory perception. The respiratory epithelium, lined with specialized cilia, acts as a first line of defense by sweeping away inhaled particulate matter and foreign substances, while the rich vascular network of the mucosal membrane conditions the air by warming and humidifying it. Deeper within the cavity, the olfactory epithelium houses specialized neurons that mediate the sense of smell, a function closely linked to overall sensory experience and well-being. Beyond these physiological roles, the nasal pathway provides a unique opportunity for drug delivery to the CNS. Unlike systemic administration, intranasal delivery bypasses the restrictive BBB and avoids the extensive metabolic degradation associated with oral routes, offering a noninvasive and efficient strategy for targeting therapeutic agents to the brain.^7–10^ The noninvasive nature of intranasal administration, together with its capacity for precise and localized drug distribution, represents a significant advancement in neuropharmacology. This approach not only enhances the efficiency of CNS drug delivery but also has the potential to improve therapeutic outcomes, minimize systemic side effects, and address pressing unmet needs in the treatment of neurological disorders.^11,12^

Among the various strategies explored to harness this potential, dendrimers have attracted considerable attention as versatile nanocarriers. Their well-defined architecture, modifiable surface functionalities, and capacity to encapsulate or conjugate therapeutic molecules make them especially suitable for enhancing intranasal delivery to the brain.^13,14^ Dendrimers are nanoscale, highly branched macromolecules with a tree-like structure that offers multiple binding sites. This unique architecture enhances their versatility and makes them an effective vehicle for delivering therapeutic agents into the brain.^15^ Their potential to overcome challenges such as the BBB and targeted delivery has been highlighted in recent studies.^16^ Poly­(amidoamine) (PAMAM) dendrimers are highly branched, symmetrical macromolecules with well-defined structures that offer significant surface functionality, making them effective for loading and delivering therapeutic agents. These mixed surfaced and positively charged nanoparticles are widely studied as nonviral delivery vectors in gene therapy, providing protection for nucleic acids against enzymatic degradation and facilitating cellular uptake, including endocytosis and endosomal escape.^17,18^ The properties of PAMAM dendrimers vary with generation, with higher generations forming larger, more globular, and densely packed structures, while lower generations exhibit open and asymmetric shapes.^19,20^ The surface chemistry plays a critical role in determining dendrimer biocompatibility and cellular interactions. For instance, PAMAM dendrimers with 100% amine surface groups exhibit high cytotoxicity due to their strong positive charge, which can disrupt cell membranes and induce cell death.^21^ In contrast, dendrimers modified with neutral groups such as hydroxyls or carboxyls demonstrate significantly improved safety profiles.^22^ Studies have reported that cationic higher-generation dendrimers can even induce intravascular coagulation or alter platelet function, while lower-generation dendrimers (G0–G3) show more favorable safety outcomes.^23,24^

Mixed-surface PAMAM dendrimers, containing hydroxyl groups as well as amine groups in their terminal branches, have been engineered to improve the balance between therapeutic efficacy and biocompatibility compared to fully amine-terminated counterparts, which are known to cause significant cytotoxicity. For example, G4–90/10 dendrimers, containing 90% hydroxyl and 10% amine surface groups, display high structural precision, are readily labeled with fluorescent dyes, and show markedly reduced toxicity, up to an 8-fold decrease in cell death compared to 100% amine-terminated PAMAM dendrimers while still maintaining sufficient cationic charge for biological interactions.^25^ Similarly, G4–70/30 dendrimers, composed of 70% hydroxyl and 30% amine termini, retain enough surface amines to enable electrostatic binding of negatively charged nucleic acids and small-molecule drugs, while further minimizing cytotoxic effects relative to pure amine dendrimers.^26^ Both formulations are capable of cellular uptake, can be conjugated to therapeutic payloads through standard bioconjugation techniques, and have been shown to cross biological barriers, such as the BBB under certain delivery conditions. Although the exact mechanisms of internalization in neuronal and glial cells remain under investigation, proposed uptake pathways include clathrin-mediated endocytosis, caveolae-mediated endocytosis, and macropinocytosis, suggesting their considerable versatility as nanocarriers for central nervous system–targeted drug delivery.^27,28^ In this study, we investigate the ability of G4 70/30 PAMAM dendrimers (Figure 1) to reach the brain following intranasal administration, aiming to evaluate their potential as safe and effective carriers for CNS-targeted therapies.

**Figure 1:**
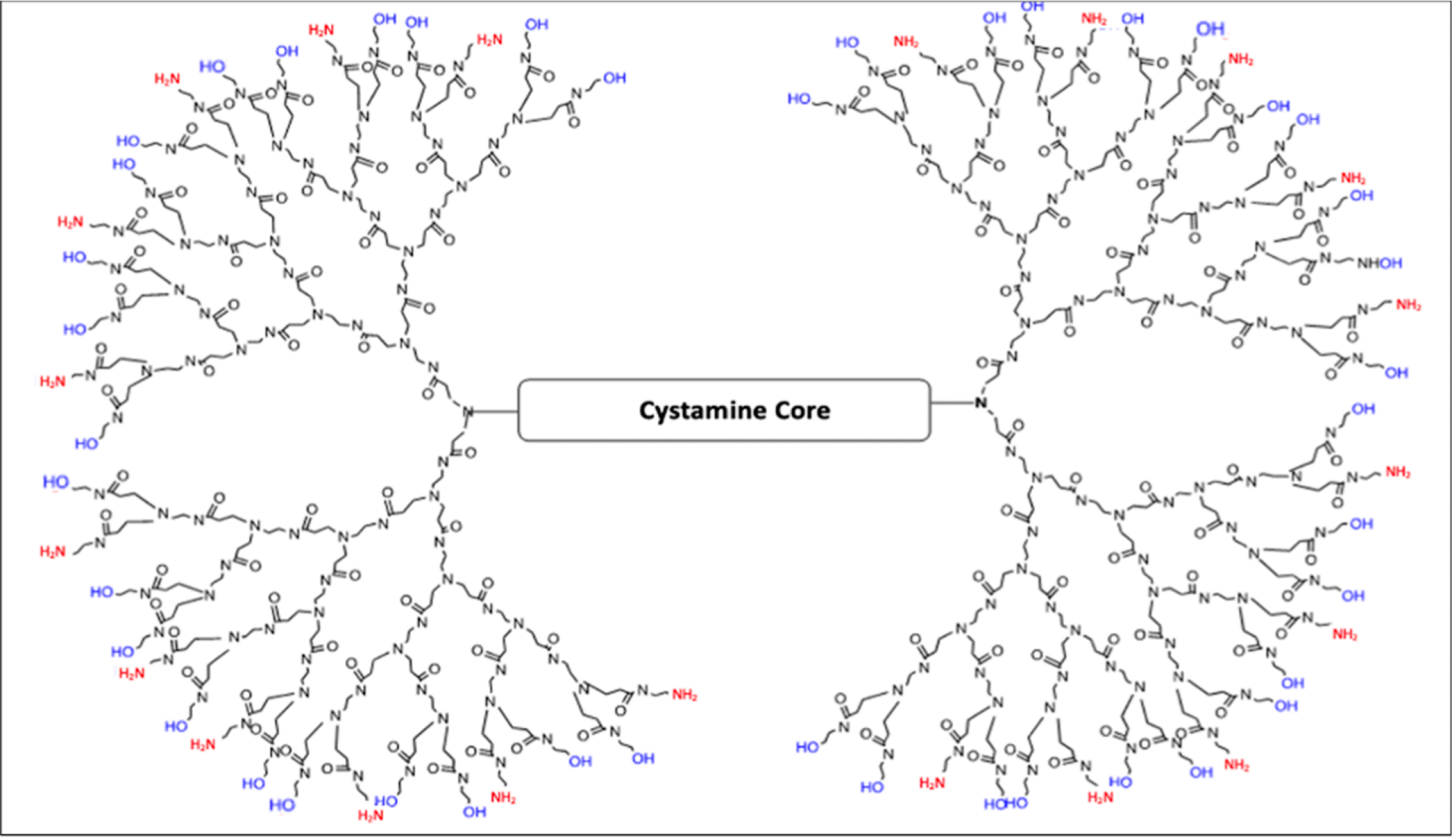
Representation of the G4–70/30 dendrimers having 70% hydroxyl groups and 30% amine groups.

## 2 Results

### 2.1 Assessment of PAMAM Dendrimer Purity and Surface Amine Density via PAGE

Polyacrylamide gel electrophoresis (PAGE) was used to assess the purity and surface composition of PAMAM dendrimers. Both G4 70/30 and G4 90/10 PAMAM dendrimers have an approximate molecular weight of 14.2 kDa. Modifying the surface amine-to-hydroxyl ratio does not substantially alter the overall molecular weight, as the changes are limited to terminal group composition rather than the dendrimer core structure. As shown in Figure 2, the migration pattern reflected the relative density of surface amines. The dendrimer with the highest proportion of amine groups (lane 1) migrated fastest, producing a sharp and distinct band, indicating high purity. In comparison, the mixed-surface dendrimers (lane 2, 70% OH/30% NH_2_) migrated more slowly, consistent with their reduced net positive charge (Figure 2). The G4–90/10 dendrimer (lane 3, 90% OH/10% NH_2_) showed the slowest migration, as expected for a dendrimer with the lowest number of surface amines. These results confirm that dendrimer migration in PAGE is directly dependent on surface amine density. Even dendrimers with predominantly hydroxyl surfaces retained measurable mobility due to protonation of internal tertiary amines under the acidic conditions used. Collectively, the PAGE analysis demonstrates the expected migration hierarchy, consistent with dendrimer surface composition and confirming the purity of the synthesized materials.

**Figure 2:**
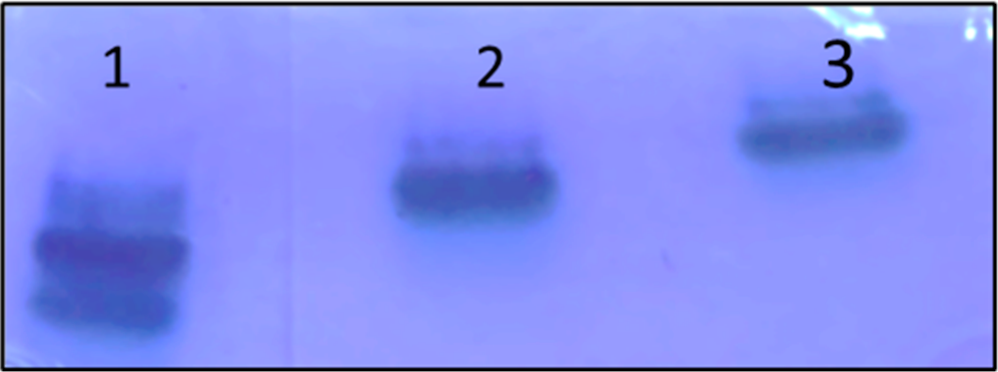
PAGE analysis of PAMAM dendrimers with varying surface compositions. The dendrimer with 100% surface amines (lane 1) exhibited the fastest migration, followed by the mixed-surface dendrimers with 30% surface amines (lane 2) and 10% surface amines (lane 3). This migration trend under acidic conditions reflects the relative surface amine density, confirming the expected correlation between dendrimer charge and electrophoretic mobility.

### 2.2 Retention Behavior of PAMAM Dendrimers by RP-HPLC

Reverse phase high-performance liquid chromatography (RP-HPLC) was used to evaluate the surface composition of the synthesized dendrimers. As shown in Figure 3, the G4 90/10 dendrimers exhibited a retention time of 9.5 min (Figure 3A), whereas the G4–70/30 dendrimers eluted earlier, at 8.5 min (Figure 3B). The longer retention time observed for G4–90/10 is consistent with the higher proportion of terminal amine groups, which engage in stronger polar and electrostatic interactions with the stationary phase. In contrast, the G4–70/30 dendrimers, with a greater density of hydroxyl groups, displayed faster elution due to their increased hydrophilicity and reduced affinity for the hydrophobic stationary phase. These findings demonstrate that RP-HPLC effectively distinguishes dendrimers according to surface functionalization and provides a complementary method for confirming dendrimer purity and chemical modification.

**Figure 3:**
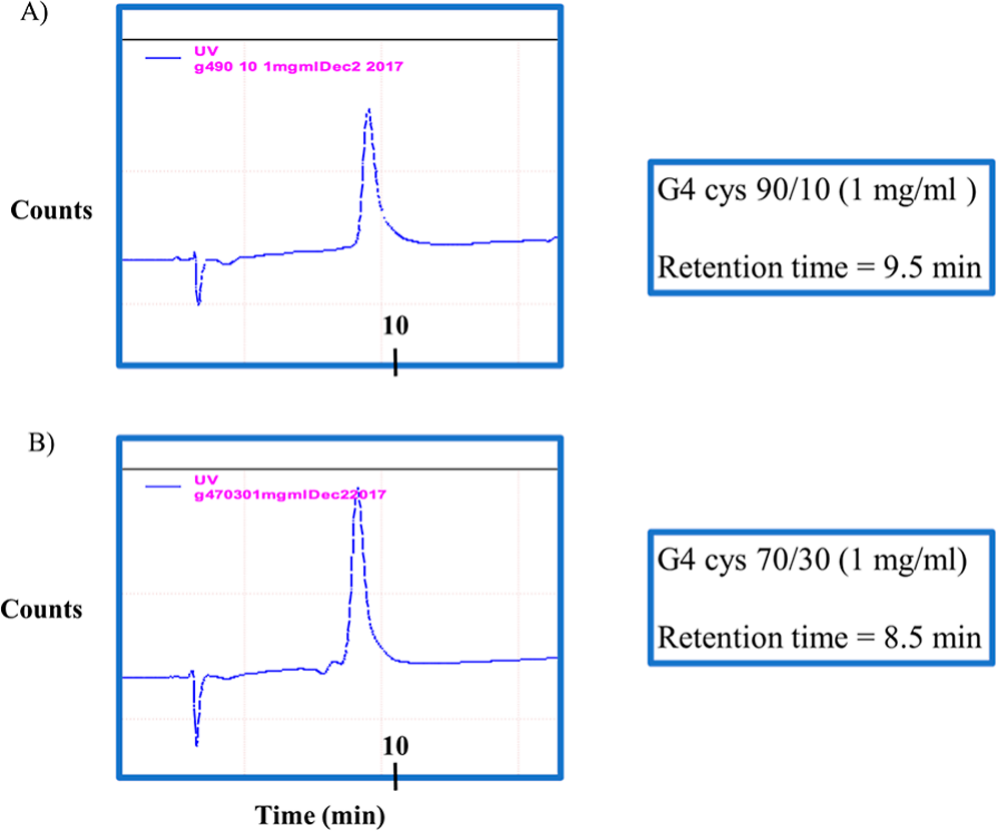
RP-HPLC of dendrimers shows different retention time between the different surface dendrimers. The retention time of G4–90/10 dendrimers is 9.5 min (A) compared to the G4–70/30 dendrimers with retention time of 8.5 min (B).

### 2.3 Proton NMR Characterization Showed Cy5.5 Dendrimer Complexation

Proton nuclear magnetic resonance (1H NMR) spectra of (A) native dendrimer, (B) Cy5.5 NHS ester, and (C) Cy5.5-conjugated dendrimer were recorded on a Bruker NMR spectrometer operating at 500 MHz using D_2_O and DMSO-*d*_6_ (Sigma-Aldrich) as solvents. Chemical shifts (δ) are evidenced in parts per million (ppm) relative to residual solvent peaks at 4.70 ppm for D_2_O and 2.51 ppm for DMSO-*d*_6_. The spectrum of the Cy5.5-conjugated dendrimer (Figure 4C) shows the emergence of a distinct aromatic resonance at ∼8.5 ppm, confirming the formation of an amide bond between the primary amine groups of the dendrimer and the NHS ester of Cy5.5. Concurrently, a marked reduction in the characteristic Cy5.5-NHS signals at ∼2.6 ppm indicates successful consumption of the NHS ester during conjugation. Importantly, the aliphatic proton resonances of the 70/30 dendrimer framework (2.2–3.8 ppm) remain clearly observable, demonstrating that the dendrimer backbone retains its structural integrity following Cy5.5 conjugation. Regarding the number of dye molecules conjugated per dendrimer, Cy5.5 labeling was performed using NHS-ester chemistry, which selectively reacts with primary amine groups and does not react with hydroxyl (−OH) groups. Therefore, Cy5.5 conjugation occurs exclusively at amine-terminated surface sites of the dendrimers. The PAMAM G4 90/10 dendrimer contains six primary amine groups. Based on a targeted 50% conjugation efficiency, approximately 2–3 surface amines are expected to be conjugated with Cy5.5 per dendrimer, according to stoichiometric calculations. The PAMAM G4 70/30 dendrimer contains 18 primary amine groups. Using a 30% conjugation ratio, approximately 4–5 surface amines are expected to carry Cy5.5 molecules per dendrimer. To confirm removal of unconjugated dye, the absorbance of Cy5.5-labeled G4–70/30 dendrimers was measured. The absorbance at ∼650 nm was minimal (*A* ≈ 0.10), indicating that free Cy5.5 had been effectively removed and that the remaining signal corresponded to dendrimer-bound dye.

**Figure 4:**
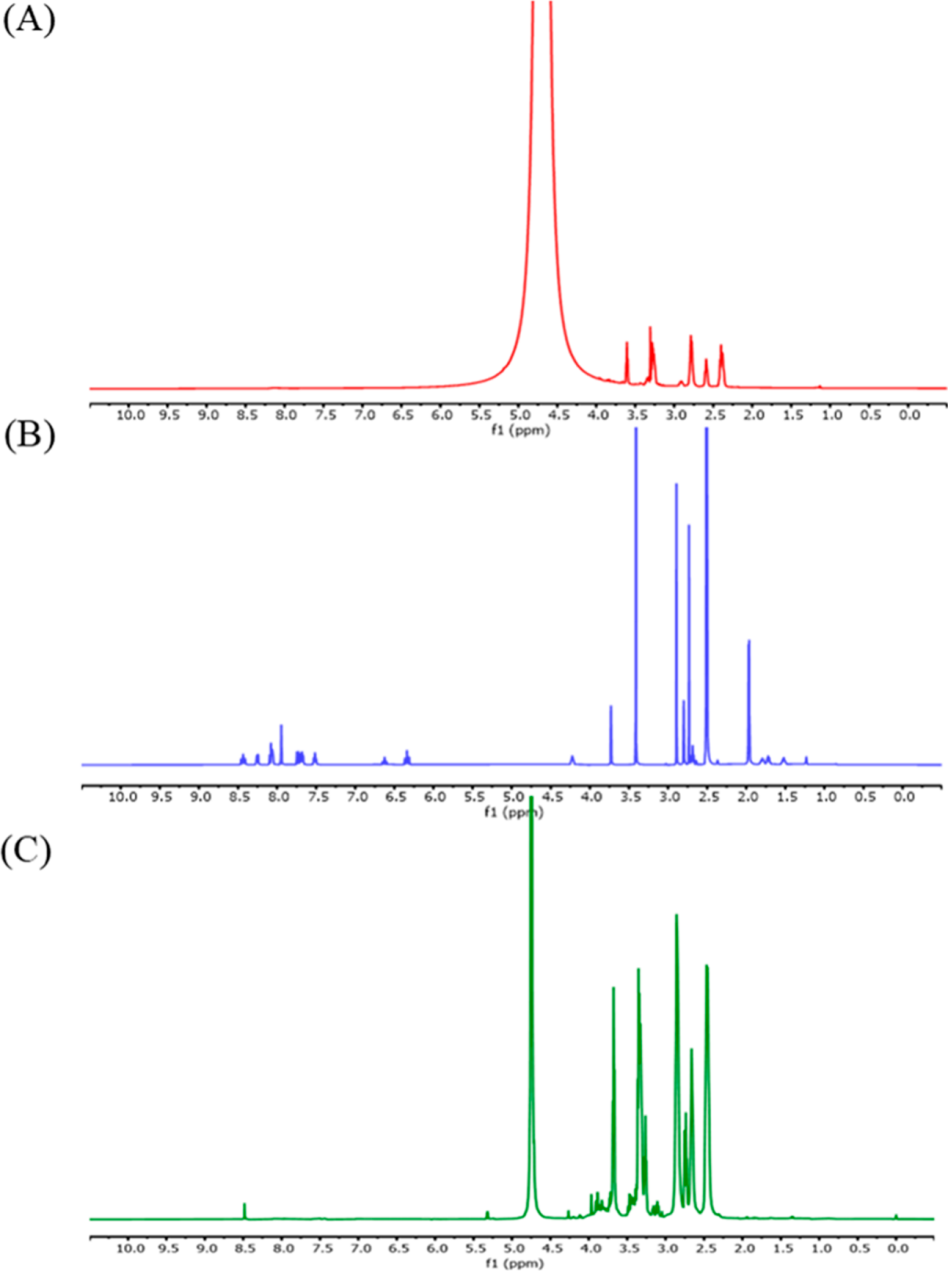
Proton NMR spectra of (A) dendrimer in D_2_O, (B) Cy 5.5 NHS ester in DMSO-*d*_6_ and (C) Cy 5.5 conjugated dendrimer in D_2_O at 500 MHz. The appearance of a strong aromatic peak at ∼8.5 ppm indicated successful amide bond formation between the primary amine groups of the dendrimer and the NHS ester of Cy 5.5. Concurrently, the characteristic Cy5.5 NHS signal at ∼2.6 ppm decreased, confirming NHS ester consumption during the conjugation reaction. The aliphatic resonances of the 70/30 PAMAM dendrimer (2.2–3.8 ppm) remained prominent, indicating preservation of the dendrimer backbone structure after functionalization.

### 2.4 Dynamic Light Scattering (DLS) Analysis Revealed that the Size Distribution by Intensity Shifted upon Cy5.5 Conjugation, Confirming Successful Coupling of the Dye to the Dendrimer

Dynamic light scattering (DLS) measurements were used to evaluate the size distribution of dendrimers before and after Cy5.5 conjugation. The unconjugated dendrimer displayed a primary peak around ∼7.024 (nm) (Figure 5A). Following Cy5.5 coupling, the size distribution by intensity showed a shift, with the major peak maintained around ∼9.051 (nm) and with increased scattering intensity, confirming successful dye attachment (Figure 5B). The persistence of the nanoscale peak alongside reduced aggregate contribution suggests that Cy5.5 conjugation did not disrupt the overall dendrimer structure while providing evidence of covalent linkage. In the DLS measurements, a secondary peak was also observed in the 1000–10,000 nm range. We attribute this signal primarily to potential bubble formation, dust particles, or other external contaminants introduced during sample preparation, as DLS is highly sensitive to large scatterers. Dendrimer dimerization due to electrostatic (charge–charge) interactions has been reported in the literature; however, such species typically appear at sizes below 100 nm. Therefore, the large secondary peak is unlikely to represent true dendrimer dimers.

**Figure 5:**
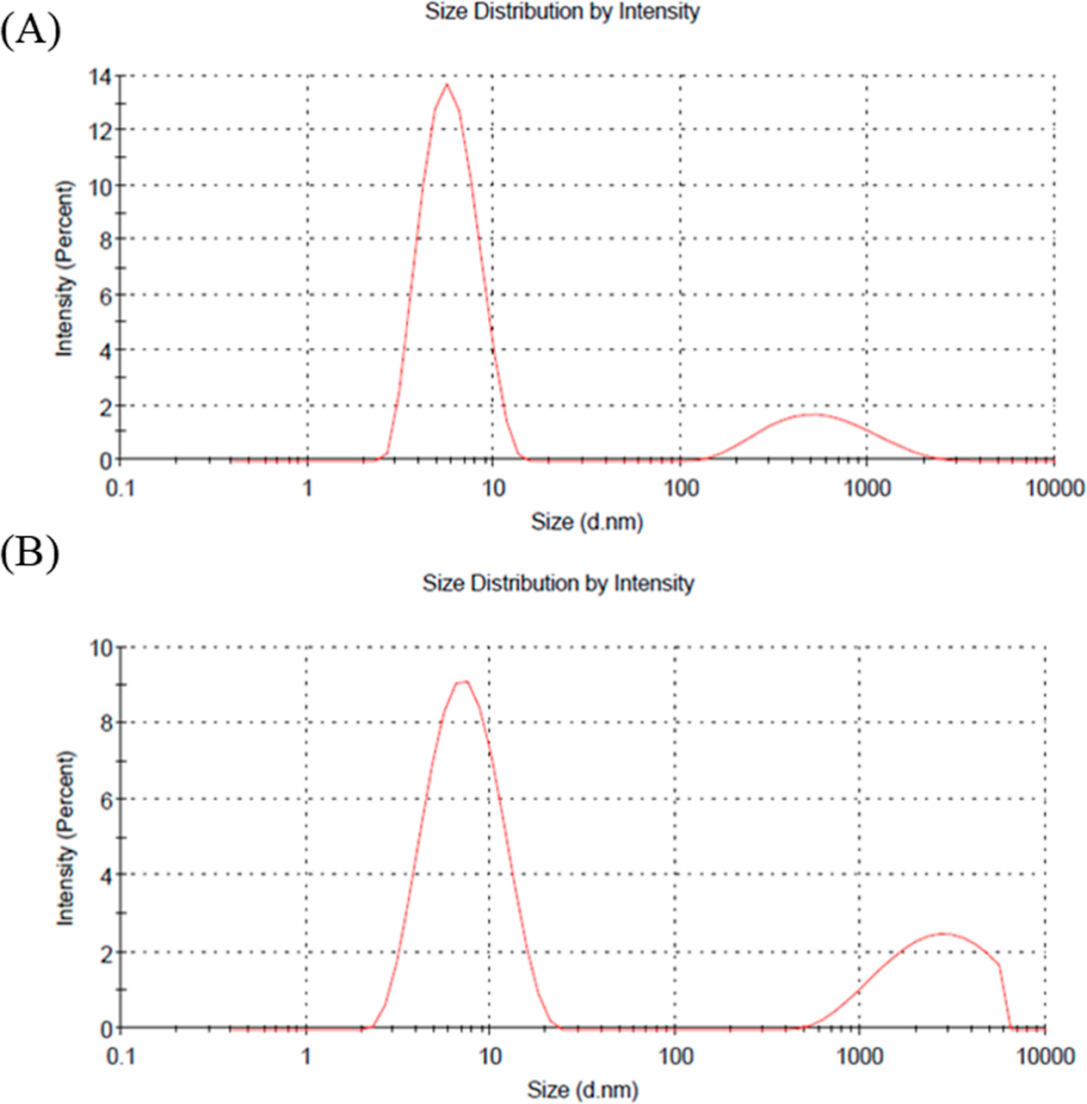
Dynamic light scattering (DLS) size distribution by intensity of dendrimers before and after Cy5.5 conjugation. (a) Unconjugated dendrimer shows a primary peak at ∼7.024 nm, corresponding to the hydrodynamic diameter of individual dendrimers, with a secondary broader peak at ∼2000–5000 nm indicating minor aggregation. (b) Cy5.5-conjugated dendrimer displays a similar primary peak at ∼9.051 nm with increased scattering intensity, confirming successful coupling of Cy 5.5 with dendrimer, while the aggregate peak is reduced compared to the unconjugated dendrimer.

### 2.5 Cy5.5 G4 70/30 PAMAM Dendrimer was Not Toxic to Primary Mouse Astrocyte Cells

The cytotoxicity of G4 70/30 PAMAM/Cy5.5 dendrimer was assessed in primary mouse astrocyte cells using the MTT assay. Cells were treated with increasing concentrations of the dendrimer (1, 2, 5, 10, 15, and 20 mg/mL), and cell viability was measured 24 h post-treatment. As shown in Figure 6, all tested concentrations-maintained cell viability above 90% relative to the untreated control. Statistical analysis indicated no significant differences between any of the treated groups and the control (*p* > 0.05), suggesting a lack of cytotoxicity across the tested dose range (Figure 6). These results demonstrate that the G4 70/30 PAMAM/Cy5.5 dendrimer is well tolerated by mouse astrocyte cells and exhibits excellent biocompatibility in vitro.

**Figure 6:**
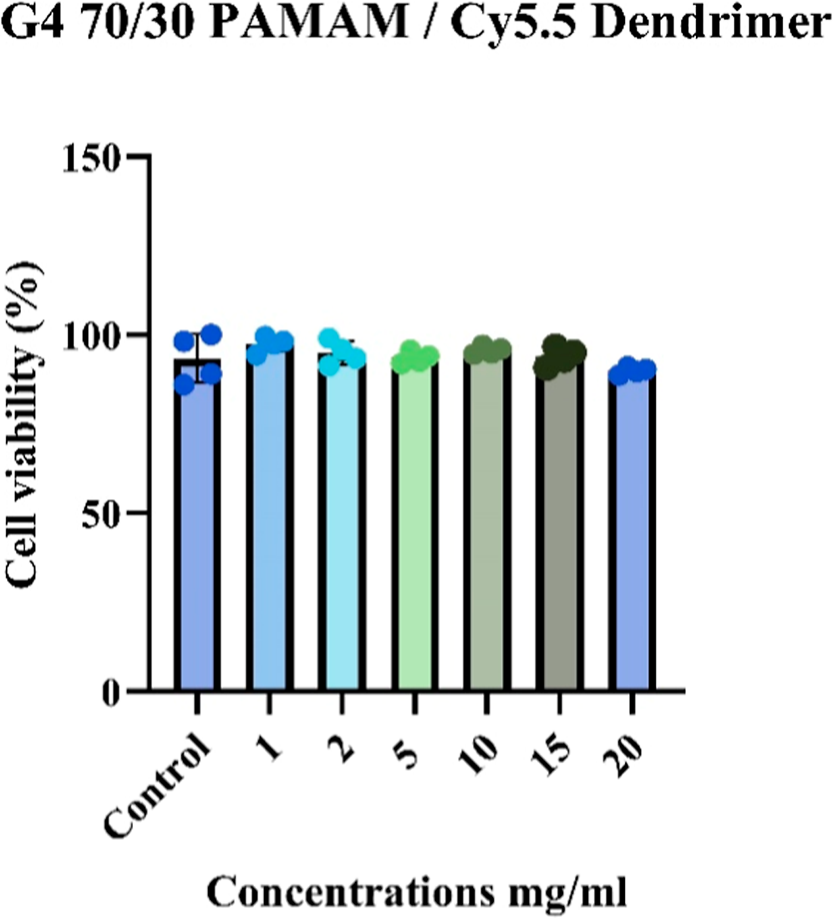
G4 70/30 PAMAM/Cy5.5 dendrimer does not induce cytotoxicity in primary mouse astrocytes. Cell viability remained above 90% for all concentrations (1, 2, 5, 10, 15, and 20 mg/mL) compared to untreated control cells. Statistical analysis showed no significant differences (ns, *p* > 0.05) between treated groups and control. These results indicate that G4 70/30 PAMAM/Cy5.5 dendrimer is biocompatible and not toxic to mouse astrocytes in vitro.

### 2.6 Presence of Cy5.5 PAMAM Dendrimers in Primary Brain Astrocytes

Following a single treatment, Cy5.5-labeled PAMAM dendrimers were readily taken up by primary brain astrocytes. As shown in Figure 7, fluorescence was observed throughout the culture, indicating dendrimer internalization across the entire astrocyte population in vitro.

**Figure 7:**
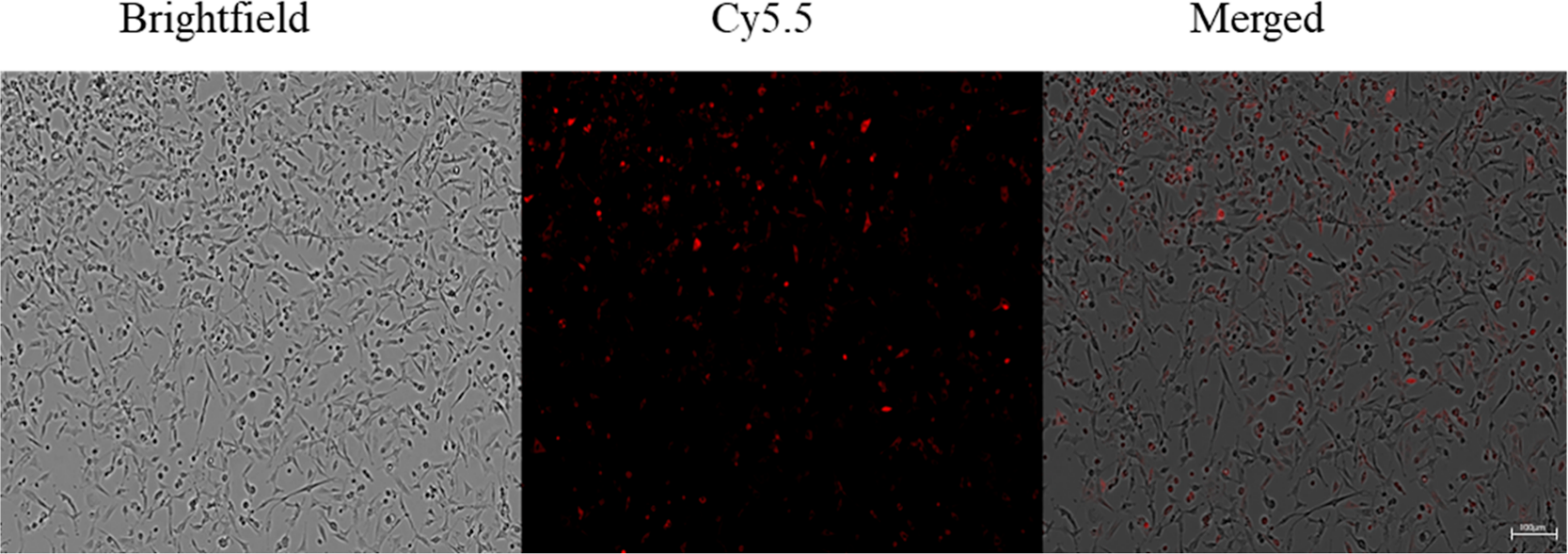
Cellular uptake of Cy5.5 by primary mouse astrocytes. First image (left) shows brightfield morphology of astrocytes, (middle) fluorescence micrograph indicating Cy5.5 signal (red), and (right) merged image confirming intracellular localization of Cy5.5 within astrocytes. The presence of red fluorescence within the cytoplasm demonstrates efficient uptake of Cy5.5 by primary mouse astrocytes. Scale bar = 100 μm.

### 2.7 Uptake of Cy5.5 Dendrimers in the Brain Following Intranasal Administration

Cy5.5-labeled dendrimers were administered to C57BL/6 mice via intranasal delivery at a concentration of 10 mg/mL. Brain uptake was evaluated at different time points, as described in the Methods section. As shown in Figure 8, a significantly higher accumulation of Cy5.5 dendrimers was observed at week 4 compared to earlier time points.

**Figure 8:**
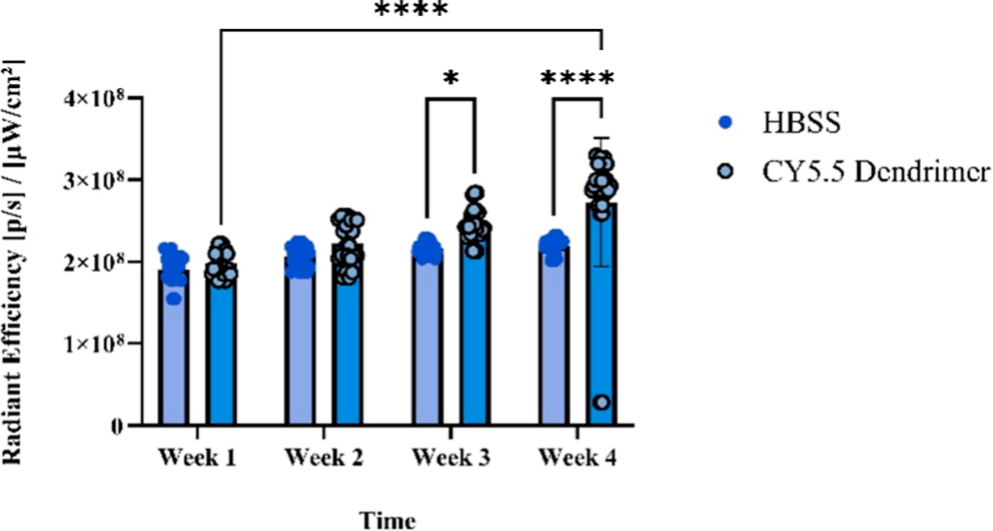
Quantification of average radiant efficiency in C57BL/6 mice administered HBSS or Cy5.5 dendrimers over 4 weeks. Data are presented as mean ± SEM. At Weeks 1 and 2, no significant difference was observed between groups (ns). By Week 3, Cy5.5 dendrimer-treated mice exhibited significantly increased fluorescence compared to HBSS (*p < 0.05). This difference was further enhanced at Week 4 (***p < 0.001). Overall, fluorescence intensity increased significantly across time points in the Cy5.5 dendrimer group (****p < 0.0001), indicating progressive accumulation of dendrimers in the brain over the experimental timeline.

### 2.8 Influence of Sex and Age on Cy5.5 Dendrimer Uptake

To determine whether sex or age influenced brain uptake of Cy5.5 dendrimers, both male and female C57BL/6 mice, as well as young and aged groups, were analyzed. Male C57BL/6 mice exhibited significantly higher fluorescence intensity compared to females, indicating greater dendrimer accumulation. In contrast, no significant differences were observed between young and aged C57BL/6 mice within each sex group (Figure 9).

**Figure 9:**
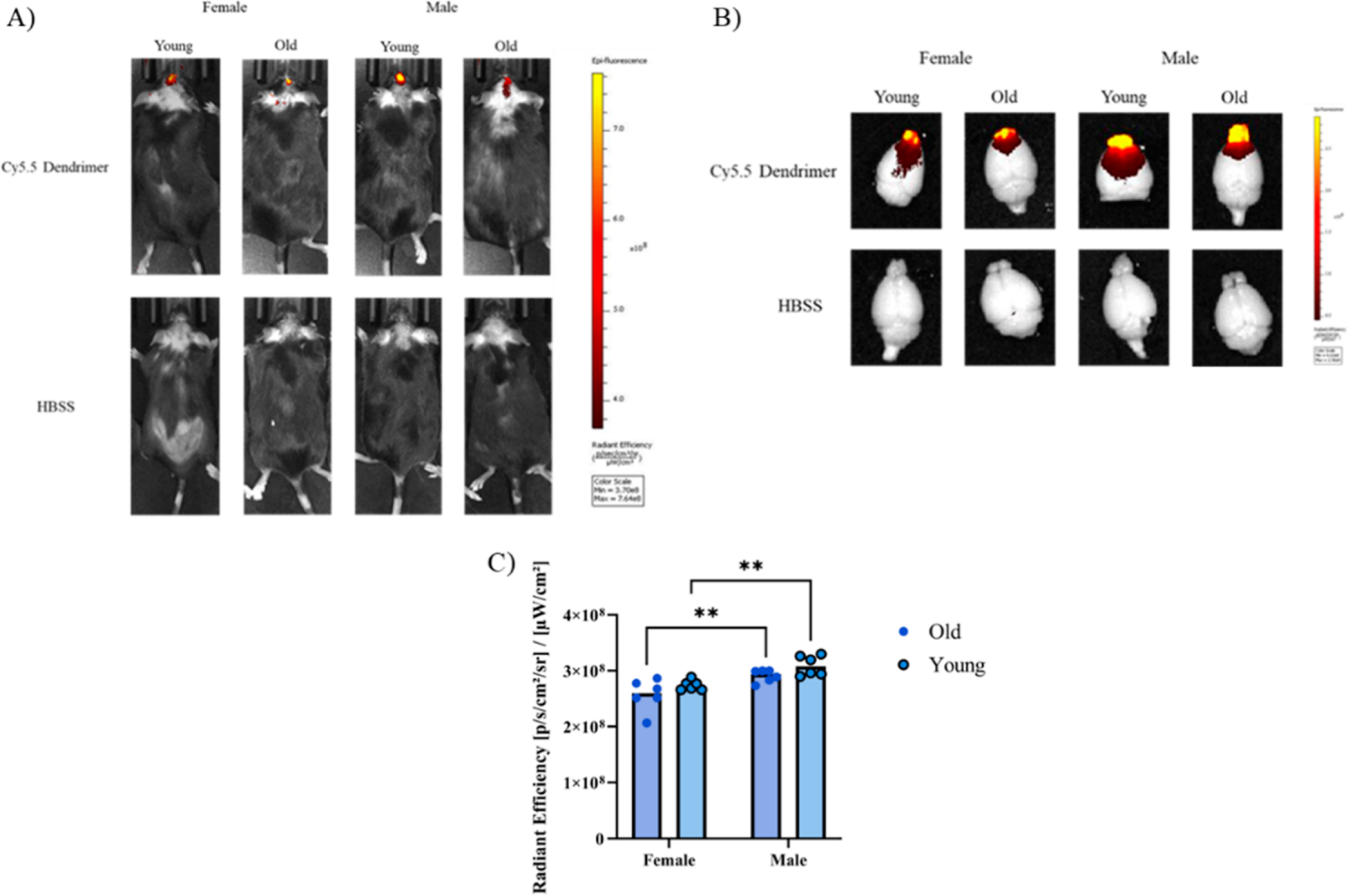
Intranasal delivery and biodistribution of Cy5.5-labeled dendrimers in C57BL/6 mice. Representative in vivo fluorescence imaging and quantitative analysis of Cy5.5 dendrimer uptake in intact C57BL/6 mice (A) and extracted brains (B). Representative IVIS images showing fluorescence signal intensity localized to the head and brain regions following intranasal administration of Cy5.5 dendrimers. The color scale represents radiant efficiency (p/s/cm^2^/sr)/(μW/cm^2^), with warmer colors indicating higher fluorescence intensity. (C) Quantitative comparison of dendrimer uptake between sexes and age groups. Male mice exhibited significantly greater fluorescence intensity compared to females (**p < 0.01), indicating enhanced dendrimer accumulation. No significant differences were observed between young and aged mice within either sex group.

### 2.9 Biodistribution of Cy5.5 PAMAM Dendrimers in Peripheral Organs

Fluorescence imaging revealed that Cy5.5-labeled PAMAM dendrimers showed minimal accumulation in peripheral organs compared to the brain. As shown in Figure 10, only low levels of fluorescence were detected in the liver indicating limited systemic absorption following intranasal administration.

**Figure 10:**
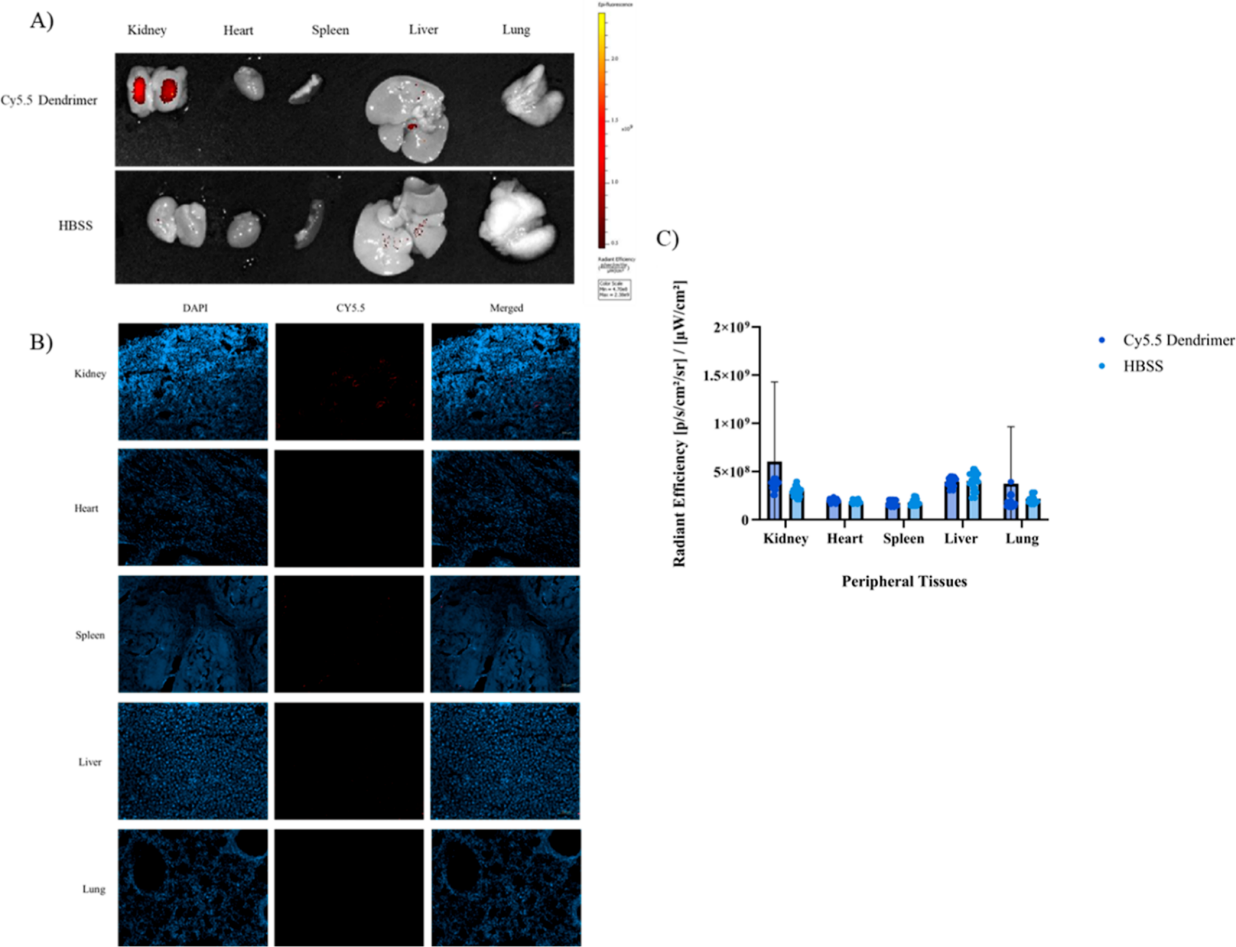
Uptake of the Cy5.5 dendrimers by the kidneys, heart, spleen, liver, and lungs, and following intranasal injections. Scale bar = 100 μm.

### 2.10 Uptake of Cy5.5-Labeled Dendrimers by Astrocytes

Colocalization analysis of Hoechst-stained nuclei with Cy5.5 dendrimers demonstrated uptake by astrocytes is shown in Figure 11. Specifically, Cy5.5 dendrimers overlapped with astrocytic marker GFAP (Figure x), indicating that systemically administered Cy5.5 dendrimers crossed the BBB following intranasal injection and was internalized by neuronal and glial cells.

**Figure 11:**
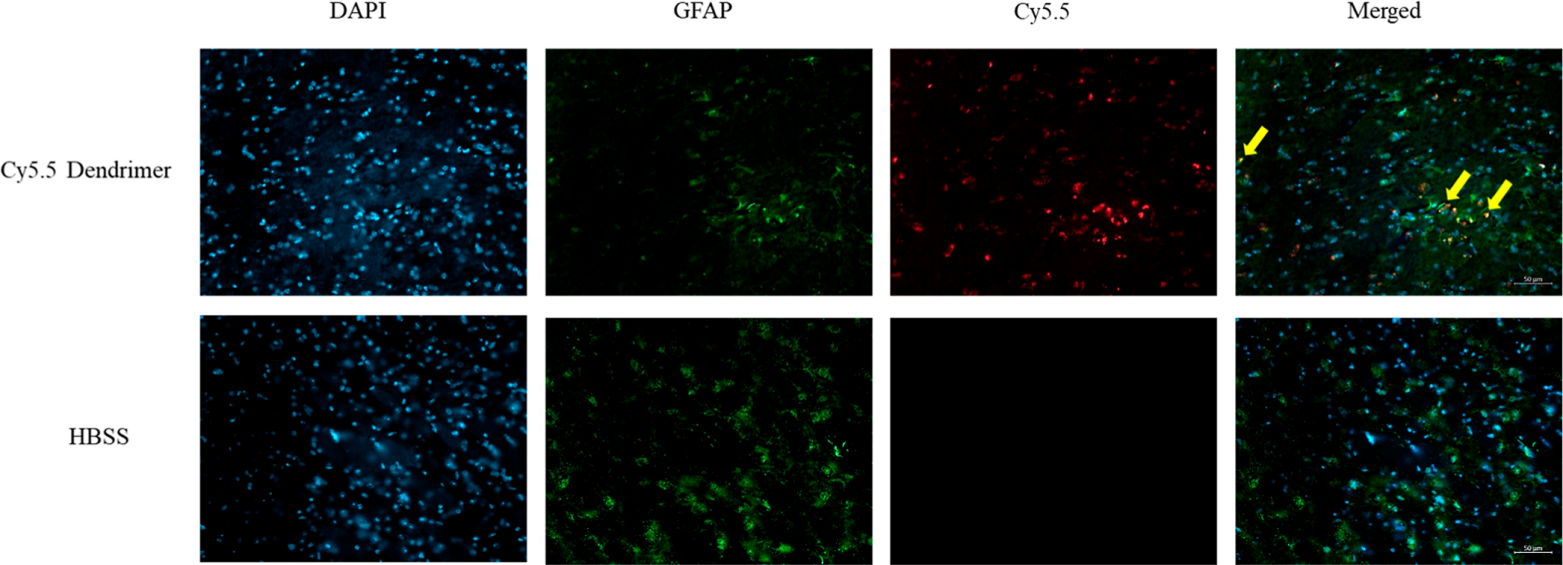
Immunofluorescence analysis showing the uptake of Cy5.5-labeled PAMAM dendrimers by astrocytes in vivo. Astrocytes were stained with GFAP Alexa Flour 488 (green), nuclei of the cells were counterstained with DAPI (blue), and dendrimers were labeled with Cy5.5 (red). The merged images (right panels) indicate colocalization of dendrimers with GFAP-positive astrocytes (yellow arrows), demonstrating effective uptake of dendrimers by astrocytes in the brain tissue. Scale bar: 50 μm.

### 2.11 Uptake of Cy5.5-Labeled Dendrimers by Neurons

As shown in Figure 12, Immunofluorescence analysis revealed that Cy5.5-labeled dendrimers colocalized with the neuronal marker NeuN, demonstrating that intranasally administered dendrimers efficiently crossed the blood–brain barrier and were internalized by neurons.

**Figure 12:**
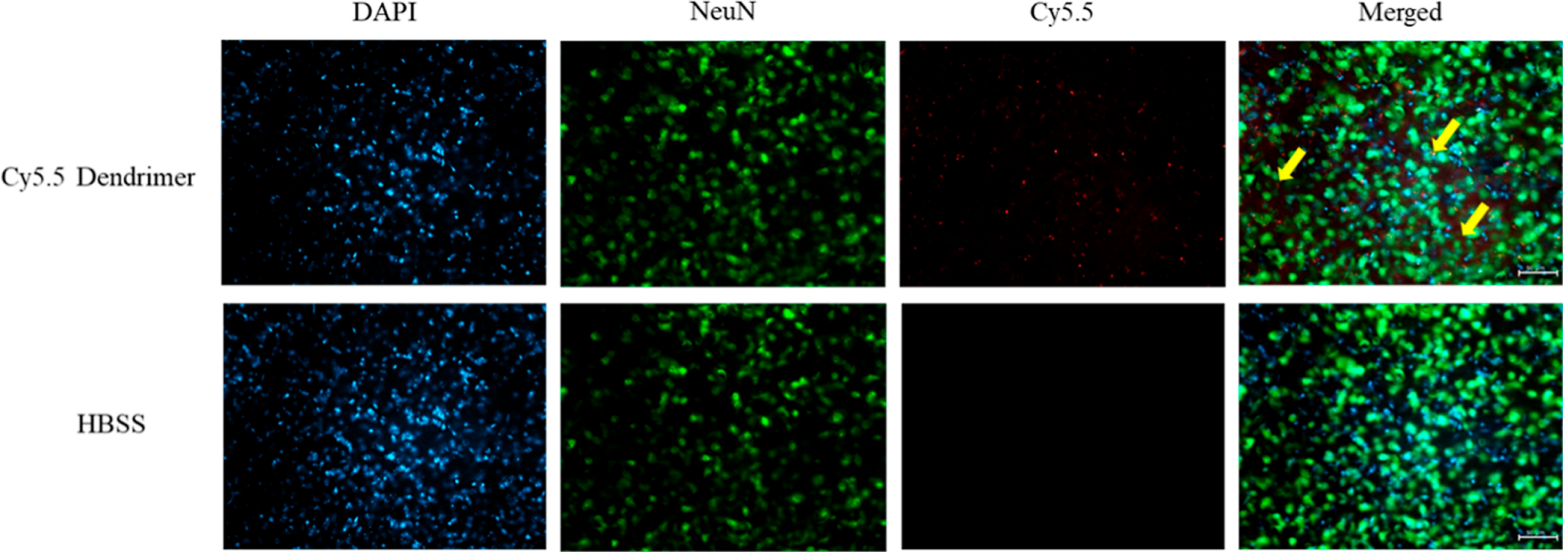
Immunofluorescence analysis of Cy5.5-labeled PAMAM dendrimer uptake by neurons in vivo. Neurons were labeled with NEUN Alexa Fluor 488 (green), cell nuclei were counterstained with DAPI (blue), and dendrimers were visualized with Cy5.5 (red). Merged images (right panels) show colocalization of dendrimers with NEUN-positive neurons (indicated by yellow arrows), confirming efficient dendrimer internalization by neuronal cells. Scale bar: 50 μm.

### 2.12 Intranasal Delivery Enables Widespread Brain Distribution of Cy5.5-Labeled Dendrimers

To evaluate the efficiency of nose-to-brain transport, we administered Cy5.5-labeled G4 70/30 PAMAM dendrimers intranasally and examined their biodistribution in brain sections. Whole sagittal brain imaging revealed robust Cy5.5 (red fluorescence) primarily along the olfactory bulb and extending into deeper structures including the cortex and striatum. Higher magnification images demonstrated that the dendrimers were widely distributed at the cellular level, with Cy5.5 signal colocalizing around DAPI-stained nuclei (Figure 13). This pattern suggests efficient neuronal and glial uptake. Importantly, the dendrimer signal was not restricted to the olfactory bulb but extended into distal regions, confirming that intranasal administration supports rapid and broad dendrimer penetration into the brain parenchyma.

**Figure 13:**
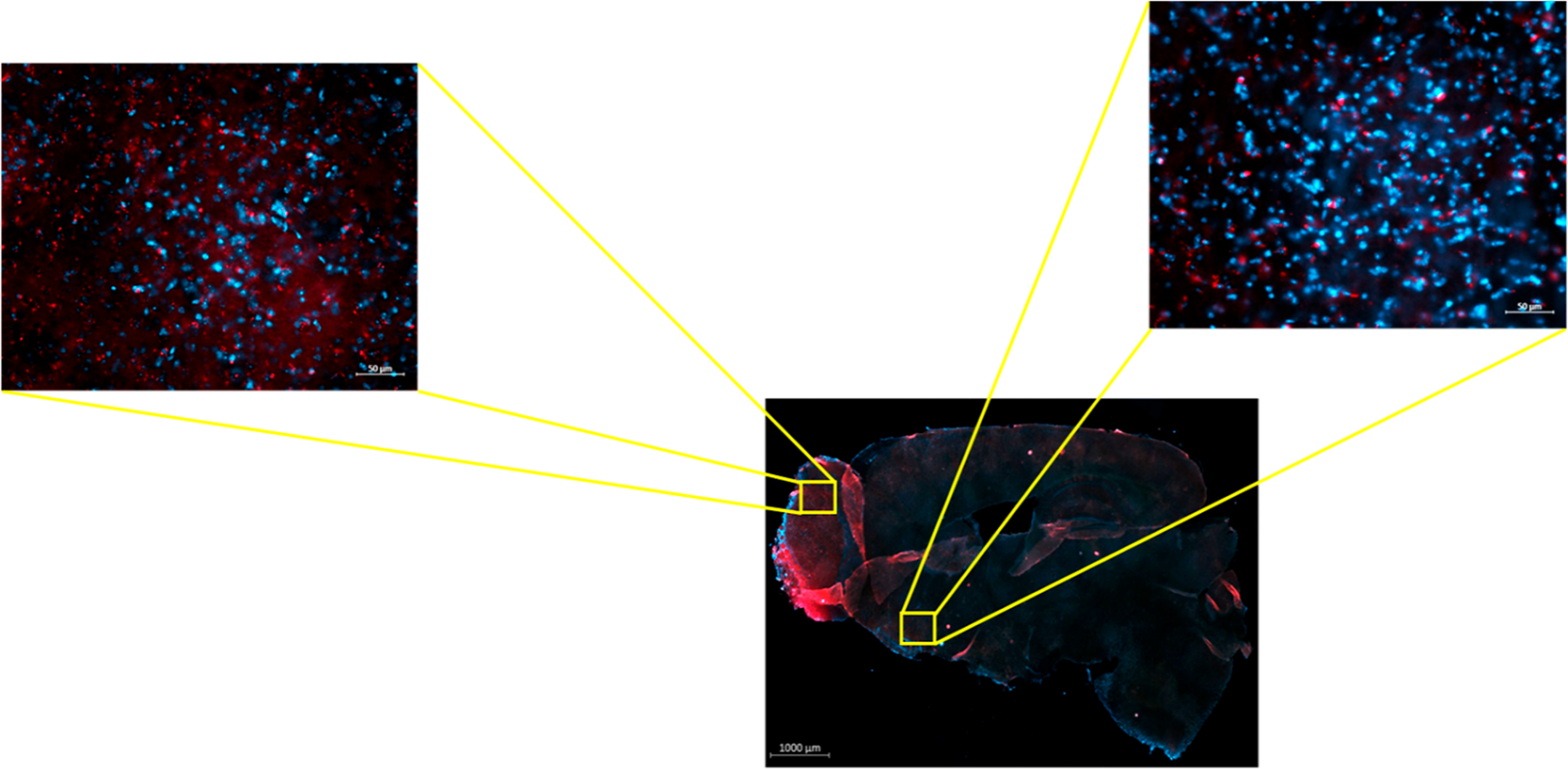
Intranasal delivery of Cy5.5-labeled G4 70/30 PAMAM dendrimers results in robust brain uptake. Representative fluorescence microscopy images of brain sagital sections following intranasal administration of Cy5.5 dendrimer (red). Nuclei were counterstained with DAPI (blue). The lower-magnification sagittal brain image shows dendrimer accumulation across multiple brain regions Scale bar: 1000 μm. Enlarged insets highlight dendrimer distribution in the olfactory bulb, cortex, and striatum, indicating efficient nose-to-brain transport Scale bar: 50 μm. Strong Cy5.5 signal was observed in perinuclear regions, consistent with neuronal and glial uptake.

These findings indicate that intranasal delivery provides a noninvasive and effective route for targeting CNS tissues with PAMAM dendrimers, bypassing systemic circulation and the blood–brain barrier.

## 3 Discussion

The blood–brain barrier (BBB) presents a major obstacle to the effective delivery of therapeutic agents to the central nervous system, as orally or intravenously administered drugs must cross this tightly regulated interface to reach their targets.^29^ An alternative strategy is intranasal delivery, which exploits the unique anatomical and physiological features of the olfactory system to provide a direct route to the brain while bypassing the BBB. Transport across the nasal olfactory and trigeminal pathways can occur via receptor, carrier, and adsorption-mediated transcytosis, thereby facilitating improved drug uptake into the brain.^30^ Poly­(amidoamine) (PAMAM) dendrimers have been widely studied as versatile drug carriers in several administration routes including intravenous,^31,32^ oral,^33^ transdermal,^34,35^ ocular,^36^ Intravenous (IV), and Intraperitoneal (IP) delivery.^37^ However, relatively little is known about their potential role as absorption enhancers in intranasal drug delivery. In this study, we demonstrated that G4 70/30 PAMAM dendrimers, with a 70% hydroxyl surface and a 30% of amines, are capable of delivering therapeutic agents to the brain via intranasal administration.

PAMAM dendrimers with mixed-surface functionalization, such as G4–70/30, offer a promising platform for CNS-targeted drug delivery due to their precise architecture, biocompatibility, and capacity for functionalization.^26^ Intranasal drug delivery enables therapeutics to reach the central nervous system (CNS) via multiple pathways, each with distinct kinetics and mechanisms. The olfactory nerve pathway provides a direct but relatively slow route, as substances absorbed at the olfactory neuron terminals are transported along axons to the olfactory bulb and deeper brain regions.^38^ In contrast, the olfactory mucosal epithelial pathway allows more rapid absorption, with drugs crossing supporting and glandular cells of the epithelium through transcellular or paracellular mechanisms, entering the cerebrospinal fluid (CSF) and brain tissue within minutes. The trigeminal nerve pathway, originating from branches that innervate the nasal epithelium, offers an additional route to the brainstem and olfactory regions, although transit along this pathway is generally slower than via the olfactory nerve.^39^ The relative contribution of each pathway depends on the physicochemical properties of the drug, its formulation, and the specific site of deposition within the nasal cavity, collectively determining the efficiency and distribution of nasal-to-brain delivery.^40,41^

Our study revealed notable sex differences in the brain distribution of G4 70/30 PAMAM dendrimers following intranasal administration. While both male and female C57BL/6 mice exhibited Cy5.5 dendrimer presence across various brain regions, males demonstrated higher overall brain uptake. This observation aligns with findings from other studies that have reported sex-specific variations in the neural and behavioral responses to intranasal compounds. For instance, research by Rilling et al. (2013) highlighted that intranasal administration of oxytocin and vasopressin elicited distinct neural activity patterns in males and females, with males showing increased brain activity in regions such as the striatum and amygdala, while females exhibited reduced or no significant changes in these areas.^42^

The observed sex differences may be attributed to several factors, including hormonal influences, anatomical variations in the nasal cavity and olfactory pathways, and differences in mucociliary clearance rates. Estrogen and progesterone, for example, have been shown to modulate the permeability of the BBB and influence the transport of substances into the brain. Additionally, structural differences in the nasal epithelium and olfactory bulb between sexes could affect the efficiency of drug uptake.^43^ Biological differences between males and females can lead to variations in drug responses. Both pharmacokinetic and pharmacodynamic differences are observed, with more extensive data available on pharmacokinetics. Generally, males have larger body sizes than females, which contributes to greater distribution volumes and faster overall clearance of many medications in males compared to females.^44^ Males generally have larger nasal cavities, which might facilitate greater drug delivery to the brain.^45^ These findings underscore the importance of considering sex as a biological variable in the development and evaluation of intranasal drug delivery systems. Moreover, we demonstrate that G4 70/30 PAMAM dendrimers can enter the brain following intranasal administration, reaching both neurons and glial cells. The dendrimers were detected in major brain regions including the olfactory bulbs, cortex, striatum, and cerebellum, confirming their ability to cross the blood–brain barrier (BBB) and gain direct access to the CNS without invasive procedures. Absorption of dendrimers through the nasal mucosa particularly via the olfactory epithelium and trigeminal nerve pathways facilitates targeted delivery to specific brain regions, with the olfactory bulbs and cortex being the initial sites of uptake due to their proximity to the nasal cavity, and further transport along neural pathways allowing distribution to deeper structures such as the striatum and cerebellum. This mechanism enables precise localization of therapeutic agents, potentially enhancing efficacy while minimizing systemic exposure.^46,47^ In line with our findings, several studies have reported that intranasal drug delivery can effectively target different brain regions, bypassing the BBB via the olfactory and trigeminal nerve pathways. For instance, a study by Lee et al. (2020) utilized labeled oxytocin administered via the intranasal route and observed its presence in specific brain regions, including the striatum and cerebellum. These findings suggest that intranasal administration can deliver substances directly to these areas of the brain.^48^ Additionally, research by Veronesi et al. (2020) highlighted the potential of intranasal delivery for targeting various brain regions. While the study did not focus exclusively on the striatum and cerebellum, it supports the concept of intranasal administration reaching multiple brain areas.^49^

Biodistribution studies following intranasal delivery revealed that Cy5.5 G4 70/30 PAMAM dendrimers accumulate primarily in the kidneys, with smaller amounts detected in the liver, and spleen. This pattern suggests efficient renal clearance, which may reduce the risk of long-term accumulation and systemic toxicity. Renal clearance is advantageous because it minimizes metabolic interactions and reduces potential toxicity; however, it also limits circulation time, which can affect therapeutic efficacy.^50^ Overall, this study highlights the potential of mixed-surface PAMAM dendrimers as versatile nanocarriers for intranasal drug delivery. The delivery method, biodistribution, low toxicity, and ability to transport cargo into neurons and glial cells of G4 70/30 PAMAM dendrimers make them promising candidates for the development of therapies targeting neurological disorders.

## 4 Methods

The present study was designed to evaluate the biodistribution and safety of G4 70/30 Cy5.5-labeled PAMAM dendrimers in the brain following repeated intranasal administration in old and young C57BL/6 mice. All experimental procedures were approved by the Institutional Animal Care and Use Committee (IACUC) at Central Michigan University under protocol #2024-497.

### 4.1 Synthesis of G4 70/30 PAMAM Dendrimers

The G4 70/30 PAMAM dendrimers were synthesized using the same protocol as previously described for the G4 90/10 PAMAM dendrimers.^51^ The general synthesis of PAMAM dendrimers involves iterative alkylation of amine groups with methyl acrylate, followed by amidation of the resulting methyl esters using a large excess of ethylenediamine. Each complete cycle of alkylation and amidation yields a new dendrimer generation, with the first cycle from the core amine producing generation 0 (G0), the second cycle generation 1 (G1), and so forth. All dendrimers used in this study were synthesized with a cysteamine core. Dendrimers were purified by dialysis with 3 kDa molecular weight cutoff (MWCO) membranes (Spectrum Laboratories, Rancho Dominguez, CA, USA) against three buffer changes of 0.9% sodium chloride, followed by overnight dialysis in deionized water. The purified products were lyophilized and diluted in HBSS at 10 mg/mL concentration for subsequent in vitro and in vivo studies.

### 4.2 Preparation of PAMAM Mixed-Surface Dendrimer *G* = 4, DAB Core, 70% OH/30% NH_2_ (G4–70/30)

PAMAM dendrimer, cystamine core, generation 3.5 with a methyl ester surface was synthesized following previously reported procedures.^26^ Briefly, PAMAM G3 (amine surface) reacted with two equivalents of methyl acrylate per terminal amine in methanol at room temperature for 48 h. Volatile components were removed, and the residue was redissolved in methanol. This process was repeated three times to eliminate unreacted methyl acrylate, yielding a clear viscous oil.

For surface modification, PAMAM G3.5 methyl ester (15.5 g, 1.24 mmol, containing 79.4 mmol ester groups, MW 12,511) in 50 mL methanol was added dropwise over 30 min to a cooled (≈8 °C), stirred solution of ethanolamine (70 mol %, 67.81 g, 1.11 mol) and ethylenediamine (30 mol %, 28.61 g, 0.476 mol), corresponding to 20 equiv per ester group. The mixture was left to react without stirring at 8 °C for 16 days, followed by equilibration to room temperature for 24 h. Complete conversion of esters was confirmed by IR spectroscopy, showing disappearance of the carbonyl stretch at 1730 cm^–1^.

Residual ethylenediamine was removed by rotary evaporation under reduced pressure. The crude product was diluted to 10% (w/v) in deionized water and subjected to dialysis (100:1 water: dendrimer) for 3–4 h with eight dialysate changes, followed by two overnight exchanges (16 h each). TLC analysis confirmed removal of ethanolamine. The dialyzed product was concentrated by rotary evaporation and repeatedly dissolved in methanol with solvent strip to remove volatiles. A 10% (w/v) methanolic solution was then fractionated using a Sephadex LH-20 column (800 g, methanol as eluent). Following collection of the void volume, 20 20 mL fractions were obtained and screened by TLC (iodine chamber). Dendrimer was detected in fractions 4–16; fractions 4–14 was pooled, concentrated, and dried under high vacuum at 300 °C to constant weight, affording 16 g (1.11 mmol, 90% yield). The final product was stored as a 15–20% (w/v) solution in methanol. In this study G4 70/30 PAMAM Dendrimer was selected because it provides sufficient surface amines for stable fluorophore/drug attachment, while maintaining a nanoscale size that favors nose-to-brain transport and astrocyte uptake. Lower generations have fewer functional groups and are cleared more rapidly, limiting their utility for brain delivery.

### 4.3 Characterization of G4 70/30 PAMAM Dendrimers and Conjugate

The structural and surface characteristics of G4 70/30 dendrimers were evaluated using acidic native polyacrylamide gel electrophoresis.^16,37^ Electrophoresis was performed on 10% native gels at 100 V for 3.5 h at 4 °C. Additional structural confirmation was obtained by 13C NMR spectroscopy (Varian Mercury 300 NMR spectrometer; proton-decoupled 13C NMR, 75 MHz field strength; relaxation delay: 1.0 s; acquisition time: 1.8 s; number of scans: 2000; samples dissolved in D_2_O).

Purity and hydrophobicity profiles were assessed by reverse-phase high-performance liquid chromatography (RP-HPLC; Hitachi HPLC system, Tokyo, Japan) using a C18 column (Varian Microsorb-MV; 250 × 4.6 mm, 300 Å) coupled to a MetaGuard 4.6 mm Microsorb 300 Å 5 μm C18 guard column. The mobile phases consisted of 0.1% aqueous trifluoroacetic acid (TFA; phase A) and 0.085% TFA in acetonitrile (phase B). Elution was performed using a linear gradient of 5–90% B over 15 min at a flow rate of 1 mL/min.

Surface amine content was quantified using the 2,4,6-trinitrobenzenesulfonic acid (TNBS) assay (TS-28997; Thermo Fisher Scientific, Waltham, MA, USA), with glycine employed to generate the calibration curve.

### 4.4 Dendrimer Purification and Complexation with Cy5.5

PAMAM dendrimers were initially dissolved in methanol and purified via rotary evaporation at 45 ± 5 °C to eliminate residual solvent. To further reduce potential cytotoxicity from methanol, dendrimers were rinsed with methanol (10× the dendrimer mass) and subjected to additional evaporation cycles. The efficiency of methanol removal was confirmed by comparing 1H NMR spectra before and after purification.

Following purification, dendrimers were chemically conjugated with Cy5.5 dye under control conditions. The successful conjugation of Cy5.5 to the dendrimer was confirmed by 1H NMR spectroscopy, which revealed characteristic peaks corresponding to both dendrimer and dye protons, indicating effective chemical conjugation.

#### 4.4.1 Particle Size and Dynamic Light Scattering (DLS)

Particle size diameter (nm) and dynamic light scattering (DLS) of the dendrimers and Cy5.5 dendrimer complexes were measured using Malvern Nano-S Zetasizer (ZEN1600, Malvern Instruments Limited, UK). Prior to measurement, purified dendrimers were diluted in HBSS buffer. The size of the Cy5.5–PAMAM dendrimer conjugate was measured by dynamic light scattering (DLS) immediately after completion of the conjugation reaction (Day 1).

## 5 In Vitro

### 5.1 In Vitro Uptake of Cy5.5 Labeled G4 70/30 PAMAM Dendrimers by the Mouse-Derived Primary Astrocyte

Primary astrocytes were extracted from the brain cortex of one-month old C57BL/6 mouse at (Aug 30, 2024, and was registered under the Central Michigan University (CMU) Institutional Animal Care and Use of Committee (IACUC) protocol #2024-497).^52^ The isolated astrocytes were cultured in DMEM supplemented with fetal bovine serum (FBS) and antibiotics (penicillin/streptomycin). Cells were incubated with Cy5.5 G4 70/30 PAMAM dendrimers at a final concentration of 10 mg/mL for 24 h. After 24 h incubation time the cells were washed twice with PBS and imaged using a Tecan Spark Cyto (Tecan).

### 5.2 Cell Proliferation Assay

MTT (3-(4,5-Dimethylthiazol-2-yl)-2,5-diphenyltetrazolium Bromide) assay is a colorimetric assay for assessing cell cytotoxicity. Astrocytes were trypsinized and counted. Then, 10 × 10^3^ per well were seeded into a 96-well plate. After 24 h, the plate was treated with a different range of 10–500 μg/mL of G4 70/30 PAMAM dendrimers. To measure the cell viability of all groups, MTT assay was conducted. For this purpose, 50 μL of MTT solution (2 mg/mL) was added to each well. Subsequently, after incubation in darkness for 4 h, the growth medium was removed, and 200 μL DMSO (dimethyl sulfoxide) was added to solve the insoluble formazan crystals and then preserved for 20 min in the incubator. In the end, the optical density (OD) was measured with an Tecan Spark Cyto on a multiscan spectrum at 570–620 nm.

## 6 In Vivo

### 6.1 Animals

All animal procedures were approved by the Institutional Animal Care and Use Committee (IACUC) at Central Michigan University (Animal Use Protocol #2024-497). A total of 48C57BL/6 mice (Jackson Laboratory, Bar Harbor, ME, USA) were used in the study. Animals were categorized according to age (young and old) and sex (male and female). The distribution of animals in each experimental group is shown in Table 1. All mice were housed under standard laboratory conditions with free access to food and water.

**Table 1:** Study Groups

animal subjects	group	sex	treatment
**C57BL/6 mice**	**old age**	**males** (*n* = 6)	**HBSS** (n = 6)
			Cy5.5 Dendrimer (n = 6)
	**old age**	**females** (*n* = 6)	HBSS (n = 6)
			Cy5.5 Dendrimer (n = 6)
	**young age**	**males** (*n* = 6)	HBSS (n = 6)
			Cy5.5 Dendrimer (n = 6)
	**young age**	**females** (*n* = 6)	HBSS (n = 6)
			**Cy5.5** Dendrimer (n = 6)

### 6.2 In Vivo Imaging System (IVIS) Protocol for Cy5.5-Labeled Dendrimer Detection in the Brain

To evaluate the brain biodistribution of Cy5.5-labeled PAMAM dendrimers following repeated intranasal administration, in vivo fluorescence imaging was performed using an in vivo Spectrum Imaging System (IVIS; Lumina LT Series III). Intranasal dosing was conducted daily for 4 weeks. The Cy5.5–PAMAM dendrimer solution was administered intranasally using a calibrated micropipette, with the solution delivered directly into the nasal cavity. A total volume of 15 μL per day of a 10 mg/mL Cy5.5-labeled dendrimer solution was administered (7.5 μL per nostril). Animals were anesthetized with 2–3% isoflurane in oxygen and positioned flat within the imaging chamber. The body temperature was maintained at 37 °C using a heated stage. IVIS imaging was performed once per week during the four-week treatment period to monitor fluorescent signal in vivo. Fluorescence was detected using excitation and emission filter sets of 675 and 720 nm, respectively, specific to Cy5.5. Imaging settings were standardized across all sessions, including a 1 s exposure time, small binning, and a f/stop of 2. Regions of interest (ROIs) were manually defined over the brain region using Living Image software (Revvity), and fluorescence intensity was quantified as average radiant efficiency (photons/sec/cm^2^/sr)/(μW/cm^2^). Background signals from untreated animals were subtracted to account for tissue autofluorescence. Plasma levels were not measured during the 1–4 week study period, as the primary objective of this study was to evaluate brain localization. Instead, Cy5.5 fluorescence was assessed in major peripheral organs, including the liver, kidney, lungs, heart, and spleen, to examine systemic distribution, off-target accumulation, and overall safety.

#### 6.2.1 Ex Vivo Imaging

At the end of the four-week treatment period, animals were perfused using PBS and 4% PFA solution in accordance with approved IACUC protocols. Brains were carefully extracted, rinsed in cold PBS, and imaged immediately using the IVIS Spectrum Imaging System under the same Cy5.5-specific excitation/emission settings (675 nm/720 nm). Imaging parameters (1 s exposure, small binning, f/stop 2) were kept consistent with in vivo settings. Fluorescence intensity was quantified by drawing regions of interest (ROIs) over the whole brain and other organs using Living Image software.

Following ex vivo imaging, organs were fixed in 4% paraformaldehyde (PFA) overnight at 4 °C, then transferred to a series of 10%, 20% and 30% sucrose solutions (Thermo Fisher Co., Ward Hill, MA, USA) until saturated, after which the organs were flash frozen with 2-methylbutane (Sigma-Aldrich, St. Louis, MO, USA). Tissues were embedded in Tissue-Tek O.C.T. Compound Embedding medium (Sakura Finetek USA, Inc., Torrance, CA, USA) and cryosectioned sagittally at 20 μm thickness using Microm cryostat (GMI Microm, Minnesota, USA).

### 6.3 Immunohistochemistry (IHC)

To assess the localization of Cy5.5-labeled PAMAM dendrimers, and to examine potential colocalization of dendrimers with neuronal and glial markers, immunofluorescent staining was performed on selected brain sections from a small cohort of dendrimer-treated animals. Neuronal nuclei were labeled using a rabbit anti-NeuN antibody (1:500; ab177487, Abcam, Cambridge, UK), while astrocytes were identified with a rabbit anti-GFAP antibody (1:500; ab7260, Abcam, Cambridge, UK). Stained sections were mounted onto glass slides, coverslipped with DAPI mounting medium, and imaged using a Zeiss Observer inverted microscope.

### 6.4 Statistical Analysis

Statistical analyses were carried out using GraphPad Prism version 10. Group differences were assessed with one-way ANOVA, followed by Tukey’s HSD posthoc tests for pairwise comparisons. Statistical significance was defined as *p* < 0.05. The selection of tests was determined by experimental design and the distribution characteristics of the data.

## 7 Conclusion

In this study, we demonstrated that G4 70/30 PAMAM dendrimers can be efficiently delivered to the brain via intranasal administration, bypassing the blood–brain barrier and targeting both neurons and glial cells. These mixed-surface dendrimers showed widespread distribution across critical brain regions, including the olfactory bulbs, cortex, striatum, and cerebellum, underscoring their potential for precise and effective CNS drug delivery. Notably, sex-specific differences in brain uptake were observed, highlighting the importance of considering biological sex in the development of intranasal therapeutics. Biodistribution analysis revealed predominant renal clearance, indicating low systemic toxicity and a favorable safety profile. Collectively, G4 70/30 PAMAM dendrimers represent a versatile and promising platform for noninvasive CNS delivery. Future investigations will explore the combination of these dendrimers with therapeutic agents, including small molecules, curcumin, and gene therapies, and assess their efficacy following intranasal administration in models of neurological disorders such as Alzheimer’s disease, glioblastoma, Huntington’s disease, and Parkinson’s disease. This strategy aims to leverage the unique properties of mixed-surface dendrimers for targeted, noninvasive CNS therapies, potentially enhancing therapeutic outcomes while minimizing systemic exposure.

### 7.1 Safety

All experiments involving animals were conducted in accordance with institutional guidelines and approved by the Institutional Animal Care and Use Committee (IACUC) Animal Use Protocol 2024-497. All cell-based experiments were performed under protocols approved by the Institutional Biosafety Committee (IBC) Registration Number: 2025-149. In this study, we used fourth-generation PAMAM dendrimers with a 70% hydroxyl and 30% amine surface composition at doses shown to be well tolerated in vivo. No unexpected hazards or adverse effects were observed in the treated animals over the course of the study. Standard laboratory safety procedures were followed when handling dendrimers and Cy5.5 dye, including the use of appropriate personal protective equipment. All chemical waste was disposed of in accordance with institutional and federal regulations.

## Abbreviations

BBB: blood–brain barrier
CNS: central nervous system
PAMAM: poly­(amidoamine)
G4 70/30: fourth-generation PAMAM dendrimer with 70% hydroxyl and 30% amine surface groups
HBSS: Hank’s balanced salt solution
Cy5.5: cyanine-5.5 fluorescent dye
IVIS: in vivo imaging system
IHC: immunohistochemistry
DLS: dynamic light scattering
NMR: nuclear magnetic resonance
PDI: polydispersity index
IACUC: Institutional Animal Care and Use Committee
IBC: Institutional Biosafety Committee
OD: optical density
PFA: paraformaldehyde
PBS: phosphate-buffered saline
DAPI: 4′,6-diamidino-2-phenylindole
GFAP: glial fibrillary acidic protein
NeuN: neuronal nuclei marker
ROI: region of interest.
